# I stay at home with headache. A survey to investigate how the lockdown for COVID-19 impacted on headache in Italian children

**DOI:** 10.1177/0333102420965139

**Published:** 2020-11-04

**Authors:** Laura Papetti, Pierfrancesco Alaimo Di Loro, Samuela Tarantino, Licia Grazzi, Vincenzo Guidetti, Pasquale Parisi, Vincenzo Raieli, Vittorio Sciruicchio, Cristiano Termine, Irene Toldo, Elisabetta Tozzi, Paola Verdecchia, Marco Carotenuto, Matteo Battisti, Angela Celi, Daniela D'Agnano, Noemi Faedda, Michela AN Ferilli, Giovanni Grillo, Giulia Natalucci, Agnese Onofri, Maria Federica Pelizza, Fabiana Ursitti, Michelangelo Vasta, Margherita Velardi, Martina Balestri, Romina Moavero, Federico Vigevano, Massimiliano Valeriani

**Affiliations:** 1 Headache Center, Department of Neuroscience, Bambino Gesù Children’s Hospital, Rome, Italy; 2 Department of Statistical Sciences, Sapienza University, Rome, Italy; 3 Headache Center, Neuroalgology Department, IRCCS Foundation “Carlo Besta” Neurological Institute, Milan, Italy; 4 Department of Human Neuroscience, Section of Child and Adolescent Neuropsychiatry, “Sapienza” University, Rome, Italy; 5 Child Neurology, Department of Neuroscience, Mental Health and Sense Organs, Faculty of Medicine and Psychology, Sapienza University, Rome, Italy; 6 Child Neuropsychiatry Unit – Ismep – ARNAS Civico, Palermo, Italy; 7 Children Epilepsy and EEG Center, PO, San Paolo ASL (Azienda Sanitaria Locale), Bari, Italy; 8 Department of Medicine and Surgery, University of Insubria and ASST dei Sette Laghi, Varese, Italy; 9 Centro Cefalee per l'età Evolutiva, Dipartimento di Salute della Donna e del Bambino, Università degli Studi, Azienda Ospedaliera di Padova, Padova, Italy; 10 Dipartimento di Medicina Clinica, Sanità Pubblica, Scienze della Vita e dell’ambiente, Università degli Studi dell’Aquila, L'Aquila, Italy; 11 Neuropsichiatria Infantile, Dipartimento di Salute Mentale e Fisica e Medicina Preventiva, Università della Campania “Luigi Vanvitelli”, Napoli, Italy; 12 Unità di Neuropsichiatroia Infantile, Dipartimento di Medicina dei Sistemi, Università Tor Vergata, Rome, Italy; 13 Neurology Unit, Department of Neuroscience, Bambino Gesù Children's Hospital, Rome, Italy; 14 Center for Sensory-Motor Interaction, Aalborg University, Aalborg, Denmark

**Keywords:** Migraine, COVID-19, lockdown, lifestyle

## Abstract

**Objective:**

The present Italian multicenter study aimed at investigating whether the course of primary headache disorders in children and adolescents was changed during the lockdown necessary to contain the COVID-19 emergency in Italy.

**Methods:**

During the lockdown, we submitted an online questionnaire to patients already diagnosed with primary headache disorders. Questions explored the course of headache, daily habits, psychological factors related to COVID-19, general mood and school stress. Answers were transformed into data for statistical analysis. Through a bivariate analysis, the main variables affecting the subjective trend of headache, and intensity and frequency of the attacks were selected. The significant variables were then used for the multivariate analysis.

**Results:**

We collected the answers of 707 patients. In the multivariate analysis, we found that reduction of school effort and anxiety was the main factor explaining the improvement in the subjective trend of headache and the intensity and frequency of the attacks (*p* < 0.001). The greater the severity of headache, the larger was the clinical improvement (*p* < 0.001). Disease duration was negatively associated with the improvement (*p* < 0.001). It is noteworthy that clinical improvement was independent of prophylaxis (*p* > 0.05), presence of chronic headache disorders (*p* > 0.05) and geographical area (*p* > 0.05).

**Conclusions:**

Our study showed that lifestyle modification represents the main factor impacting the course of primary headache disorders in children and adolescents. In particular, reduction in school-related stress during the lockdown was the main factor explaining the general headache improvement in our population.

## Introduction

On 30 January, the Italian National Institute of Health confirmed the first two cases of COVID-19 infection in Italy. The infection spread to our country, especially in the North, but it involved also other regions. For this reason, on 4 March the government ordered the closure of schools and universities throughout Italy, while on 8 March Lombardy and other provinces of northern Italy became isolated “red zones”. On 10 March, the Italian Prime Minister announced that the restriction measures were to be extended to the whole country. According to the new rules, summed up in the hashtag #istayathome (#iorestoacasa in Italian), people could go out of their home only for proven necessity (the so-called ‘lockdown’). Lockdown measures had significant economic, health and lifestyle implications. As for children and adolescents, an important change was the interruption of school activities with the start of online lessons.

Several studies have emphasized the role of different risk factors for migraine in children (1,2). Dysfunctional family situation, school stress, anxiety, and insufficient leisure time have been associated with migraine onset and severity (3). In children, migraine tends to have a seasonal trend during the year, probably in relationship to school attendance with an increased risk of chronification in the winter months, when school activities are intensified (4).

The COVID-19 pandemic was a global emergency that generated both individual and collective psychological reactions and a source of stress (5) that could affect children with primary headache diseases (6). From this point of view, lockdown represented an extreme condition in which the effect of different environmental factors and stressful conditions on headache disorders could be emerging.

The present multicenter study aimed at investigating how children and adolescents with primary headache disorders were affected by stress and lifestyle changes secondary to the COVID-19 lockdown.

## Methods

### Subject recruitment

**Figure 1.**
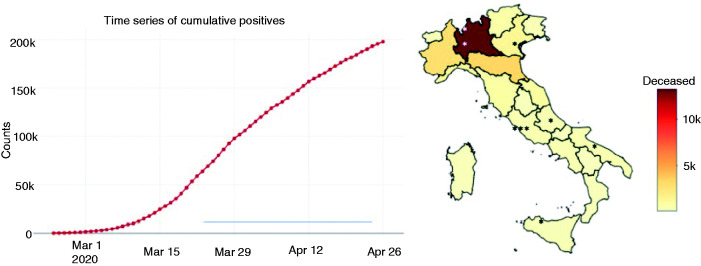
(a) Trend of infections for COVID-19 in Italy during the period of submission of questionnaire (blue line). (b) Geographical distribution of deaths for COVID-19. Asterisks show the locations of the headache centers participating in the study.

The multicenter study is based on the administration of an online questionnaire to patients, aged between 5 and 18 years (scholar age), and their parents. Patients were recruited from nine pediatric headache centers scattered throughout the Italian national territory (Figure 1). Patients were selected from the centers’ telephone books and mailing lists. Only patients with a diagnosis of migraine, with or without aura, or tension-type headache according to the ICHD-3 criteria (7) were included. Patients with other primary headache disorders, such as paroxysmal migraine and cluster headache, were excluded. Moreover, included patients had to have a headache history of at least 1 year and have visited the referral headache center in the last 6 months.

Since prophylaxis medications typically have a latency of about 3 weeks before they can take effect, only therapies that started from 3 months to 3 weeks before the compilation of the diary were considered as potentially effective. Similarly, a possible headache worsening following a recent discontinuation of a prophylactic drug was considered only if the drug had been interrupted in the 4 weeks prior to completing the questionnaire. Patients who reported a frequency equal to or greater than 15 attacks per month in the 2 months prior to the lockdown were diagnosed as having possible chronic migraine or chronic tension-type headache according to the ICHD3 criteria (7). Taking into account the average of “headache attack frequency before lockdown” and “monthly assumption of drugs for the attack before lockdown”, we built a synthetic “headache severity” index.

Towns of residence of patients have been grouped according to the macro-areas “North”, “Center”, “South and islands”.

**Figure 2.**
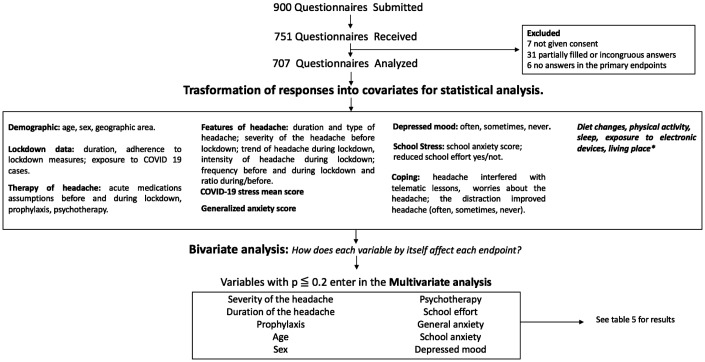
Study design. *Results concerning these variables are not discussed because they failed to show significance in multivariate analysis. Severity: Average of headache attack frequency before lockdown and monthly consumption of drug for attacks before lockdown.

Parents were asked for informed consent as well as subjects over the age of 14 were asked to give their consent. For patients under the age of 14, an assent was requested for the completion of the survey. The study was approved by the local Ethics Committee of the Bambino Gesù Children’s Hospital. The design of the study is shown in Figure 2.

### Questionnaire structure

The link to the questionnaire was sent to the patients’ families via email or via mobile phone. The questionnaire remained available online from 27 March to 20 April. The trend of infections and deaths for COVID-19 during this period is shown in Figure 1 (8). Questions were sorted to allow a gradual transition across eight topics. Some topics were explored both with questions addressed to patients and their parents. In the questionnaire, the pre-lockdown period referred to the previous 2 months of January and February 2020. The topics are summarized below: i) Demographic features; ii) lockdown information; iii) features of headache ((a) duration of headache from the first attacks; (b) type of headache disorder according to diagnosis received by the reference headache center; (c) a general judgment on the “trend of the headache” during the lockdown; (d) variation in the intensity of the attacks; and (e) the number of attacks per month in the 2 months before the lockdown and during the lockdown); iv) therapy for headache, including drugs for attacks and prophylaxis; v) anxiety about COVID-19; vi) general mood (anxiety and depressed mood) (9,10); vii) school anxiety and viii) positive coping abilities (for more details see Supplemental material and Table 1).

**Table 1.** Results from bivariate analysis for the endpoints “trend of headache”, “intensity of attacks” and “frequency of headache attacks”.

Covariates	Total patients n = 707	Improved trend Total, 323 (46%)	Stable trend Total, 277 (39%)	Worsened trend Total, 107 (15%)	Trend *p*-value	Reduced intensity Total, 270 (38%)	Stable intensity Total, 34 (48%)	Increased intensity Total, 94 (13%)	Intensity *p*-value	Frequency before the lockdown	Frequency during the lockdown	Log ratio of frequency (during/before)	Frequency *p*-value
Demographic features													
	Mean (SD)	Mean (SD)	Mean (SD)	Mean (SD)		Mean (SD)	Mean (SD)	Mean (SD)		Rho index	Rho index	Rho index	
Age in years	12.5 (3.4)	12.1 (3.3)	12.5 (3.6)	13.7 (3.2)	<0.001	11.8 (3.4)	12.7 (3.4)	13.7 (3.4)	<0.001	0.17	0.3	0.07	*p* < 0.05
	Number (%)	Number (%)	Number (%)	Number (%)		Number (%)	Number (%)	Number (%)		Mean (SD)	Mean (SD)	Mean (SD)	
Sex													
Female	425 (60)	170 (40)	179 (42)	76 (18)	<0.001	137 (32)	219 (52)	69 (16)	<0.001	8.21 (8.1)	6.02 (7.4)	−0.37 (0.8)	Ns
Male	282 (40)	153 (54)	98 (35)	31 (11)	<0.001	133 (47)	124 (44)	25 (9)	<0.001	6.14 (6.3)	3.56 (5.4)	−0.59 (0.8)	Ns
Geographic area													
North	122 (17)	51 (41)	47 (38)	24 (21)	Ns	37 (30)	69 (56)	16 (14)	Ns	9.15 (9.5)	7.2 (6)	−0.5 (0.8)	Ns
Center	458 (65)	221 (48)	174 (38)	63 (14)	Ns	185 (40)	217 (47)	56 (13)	Ns	6.74 (6.8)	4.34 (9)	−0.31 (0.8)	Ns
South and islands	127 (18)	51 (40)	56 (44)	20 (16)	Ns	48 (38)	57 (45)	22 (17)	Ns	7.98 (7.63)	5.48 (6.3)	−0.44 (0.8)	Ns
Lockdown information													
	Mean (SD)	Mean (SD)	Mean (SD)	Mean (SD)		Mean (SD)	Mean (SD)	Mean (SD)		Rho index	Rho index	Rho index	
Lockdown duration in days	33.5 (9)	33.3 (9.1)	32.6 (8.8)	34.1 (9)	Ns	33.1 (9)	34.6 (9)	35.1 (9)	Ns	0.15	0.16	0.07	Ns
	Number (%)	Number (%)	Number (%)	Number (%)	Ns	Number (%)	Number (%)	Number (%)	Ns	Mean (SD)	Mean (SD)	Mean (SD)
Exposure to COVID 19 cases													
No	684 (97)	314 (46)	266 (39)	104 (15)	Ns	263 (38)	334 (48)	78 (14)	Ns	8.4 (7.1)	6.07 (7.9)	−0.42 (0.8)	Ns
Yes	23 (3)	9 (40)	11 (47)	3 (13)	Ns	7 (31)	6 (26)	10 (43)	Ns	7.4 (6.3)	3.33 (3.4)	−0.55 (0.8)	Ns
	Number (%)	Number (%)	Number (%)	Number (%)		Number (%)	Number (%)	Number (%)		Mean (SD)	Mean (SD)	Mean (SD)	
Adherence to lockdown													
Yes	692 (97)	316 (45)	269.(39)	107 (16)	Ns	265 (38)	335 (48)	92 (14)	Ns	8.1 (8.4)	7.6 (6)	−0.6 (0.8)	Ns
No	12 (2)	5 (41)	7 (59)	0 (0)	Ns	3 (25)	7 (58)	2 (17)	Ns	7.6 (6.8)	5.3 (8)	−0.4 (0.8)	Ns
Partially	3 (1)	2 (66)	1 (34)	0 (0)	Ns	2 (66)	1 (34)	0 (0)	Ns	8.3 (6.7)	6.4 (5.3)	−0.5 (0.8)	Ns
Features of headache													
	Mean (SD)	Mean (SD)	Mean (SD)	Mean (SD)		Mean (SD)	Mean (SD)	Mean (SD)		Rho Index	Rho Index	Rho Index	
Duration of headache since onset (months)	39.6 (32)	34.1 (27)	43.1 (32)	47.1 (40)	<0.001	33.1 (27.4)	44 (32)	42.3 (39.6)	<0.001	0.03	0.16	0.18	<0.001
Severity score	6.12 (5.8)	6.64 (5.9)	6.07 (6.1)	4.71 (3.9)	<0.001	5.92 (5.3)	6.3 (6.1)	5.99 (5.5)	<0.05	0.96	0.56	−0.23	<0.001
Number of attacks per month													
Mean before lockdown	7.4 (7.5)	7.8 (7.4)	7.5 (8.4)	5.7 (5.2)		7 (6.9)	7.6 (7.9)	7.7 (7.8)	Ns	–	–	–	–
Mean during the lockdown	5 (6.8)	2.1 (2.9)	6.1 (7.4)	11.2 (8.4)	<0.001	1.93 (2.8)	6.03 (7.2)	10.4 (8.6)	Ns	–	–	–	–
Log ratio	−0.46 (0.8)	−1 (0.59)	−0.21 (0.47)	0.63 (0.56)	<0.001	−1.01 (0.63)	−0.24 (0.66)	0.34 (0.68)	Ns	–	–	–	–
	Number (%)	Number (%)	Number (%)	Number (%)		Number (%)	Number (%)	Number (%)		Mean (SD)	Mean (SD)	Mean (SD)	
Type of headache													
Episodic Mwoa	365 (52)	157 (43)	146 (40)	62 (17)	Ns	150 (41)	166 (45)	49 (14)	Ns	6.77 (6.82)	4.73 (6.43)	−0.45 (0.8)	p < 0.05
Episodic Mwa	27 (4)	12 (44)	12 (44)	3 (12)	Ns	11 (41)	15 (55)	1 (4)	Ns	3.11 (3.95)	1.74 (2.16)	−0.39 (0.8)	p < 0.05
Tension-type headache	198 (28)	96 (49)	71 (36)	31 (15)	Ns	70 (35)	102 (51)	26 (13)	Ns	9.88 (9.43)	8.25 (10.3)	−0.46 (0.6)	Ns
Chronic migraine	117 (16)	58 (49)	48 (41)	11 (10)	Ns	39 (33)	60 (51)	18 (16)	Ns	9.32 (8.88)	6.11 (7.5)	−0.48 (0.9)	p < 0.05
Intensity of attacks													
Lower	270 (38)	230 (85)	37 (13)	3 (2)	Ns	–	–	–	–	5.8 (5.2)	1.8 (5.2)	−0.65 (0.8)	p < 0.05
Stable	343 (49)	87 (25)	213 (62)	43 (13)	Ns	–	–	–	–	6.2 (4.6)	5.8 (4.6)	−0.42 (0.8)	Ns
Greater	94 (13)	6 (8)	27 (28)	61 (64)	Ns					9.3 (8.3)	6.3 (8.3)	−0.56 (0.6)	p < 0.05
Therapy of headache													
	Mean (SD)	Mean (SD)	Mean (SD)	Mean (SD)		Mean (SD)	Mean (SD)	Mean (SD)		Rho index	Rho index	Rho index	
Intake of acute medications before lockdown: no/month	4.9 (5.1)	5.4 (5.5)	4.6 (5.1)	3.7 (3.8)	<0.001	4.8 (4.8)	5.07 (5.5)	4.31 (4.47)	Ns	0.71	0.39	−0.2	Ns
Intake of acute medications during lockdown: no/month	2.8 (4.4)	1.2 (1.7)	3.6 (5.1)	5.6 (5.7)	<0.001	1.06 (1.65)	3.54 (5.4)	4.87 (3.9)	<0.001	0.31	0.65	0.47	Ns
										Mean (SD)	Mean (SD)	Mean (SD)	
Prophylactic drugs	104 (15)	38 (36)	49 (47)	17 (17)	Ns	25 (24)	60 (57)	19 (19)	Ns	11.4 (9.3)	7.4 (7.5)	−0.45 (0.75)	Ns
Psychotherapy	34 (5)	15 (45)	12 (35)	7 (20)	Ns	13 (38)	12 (36)	9 (26)	Ns	8.5 (7.5)	5.5 (7)	−0.453 (0.8)	Ns
COVID-19 stress	Mean (SD)	Mean (SD)	Mean (SD)	Mean (SD)		Mean (SD)	Mean (SD)	Mean (SD)		Rho index	Rho index	Rho index	
COVID 19 anxiety score	0.11 (0.7)	0.08 (0.65)	0.11 (0.68)	0.23 (0.88)	<0.001	0.12 (0.67)	0.04 (0.69)	0.35 (0.82)	Ns	−0.01	0.02	0.01	Ns
General Mood													
	Mean (SD)	Mean (SD)	Mean (SD)	Mean (SD)		Mean (SD)	Mean (SD)	Mean (SD)		Rho index	Rho index	Rho index	
Mean generalized anxiety score	0.05 (0.8)	−0.06 (0.8)	0.04 (0.8)	0.39 (0.8)	<0.001	−0.05 (0.82)	0.00 (0.78)	0.48 (0.85)	<0.001	0.08	0.18	0.12	<0.001
	Number (%)	Number (%)	Number (%)	Number (%)		Number (%)	Number (%)	Number (%)		Mean (SD)	Mean (SD)	Mean (SD)	
Deflected mood													
Often	49 (7)	13 (26)	20 (40)	16 (34)	<0.001	12 (25)	21 (42)	16 (33)	<0.001	7.92 (7.9)	7.2 (7.5)	−0.1 (0.6)	<0.001
Sometimes	356 (50)	156 (43)	143 (40)	57 (16)	<0.001	123 (35)	173 (49)	56 (16)	<0.001	7.58 (7)	5.2 (6.3)	−0.4 (0.8)	<0.001
Never	302 (43)	154 (50)	114 (38)	34 (12)	<0.001	131 (43)	149 (49)	22 (8)	<0.001	7.06 (8)	4.55 (7.1)	−0.5 (0.9)	<0.001
School stress													
	Mean (SD)	Mean (SD)	Mean (SD)	Mean (SD)		Mean (SD)	Mean (SD)	Mean (SD)		Rho index	Rho index	Rho index	
School anxiety score	0.12 (0.7)	−0.13 (0.6)	0.16 (0.6)	0.81 (0.7)	<0.001	−0.17 (0.59)	0.2 (0.73)	0.68 (0.75)	<0.001	0.21	0.5	0.37	Ns
	Number (%)	Number (%)	Number (%)	Number (%)		Number (%)	Number (%)	Number (%)		Mean (SD)	Mean (SD)	Mean (SD)	
Telematic lessons	625 (88)	282 (45)	244 (40)	99 (15)	Ns	240 (38)	323 (51)	62 (11)	Ns	6.7 (5.1)	4.5 (4)	−0.18 (0.71)	Ns
Reduced school effort	350 (50)	215 (61)	119 (34)	20 (5)	<0.001	191 (54)	144 (41)	19 (5)	<0.001	7.6 (7.8)	3.5 (5.1)	−0.73 (0.8)	<0.001
Coping													
	Number (%)	Number (%)	Number (%)	Number (%)		Number (%)	Number (%)	Number (%)		Mean (SD)	Mean (SD)	Mean (SD)	
Headache interfered with telematic lessons													
Often	3 (1)	0 (0)	2 (33)	1 (67)	<0.001	0 (0)	2 (67)	1 (33)	<0.001	3.3 (1.2)	3.67 (0.6)	0.09 (0.38)	Ns
Sometimes	128 (18)	31 (24)	48 (38)	49 (38)	<0.001	20 (16)	70 (55)	38 (29)	<0.001	10.3 (9)	10.3 (8)	0.02 (0.8)	Ns
Never	576 (81)	292 (51)	227 (39)	57 (10)	<0.001	250 (43)	271 (47)	55 (10)	<0.001	6.75 (7)	3.9 (5.8)	−0.57 (0.76)	Ns
Worries about headache													
Often	125 (18)	9 (7)	61 (48)	55 (45)	<0.001	7 (6)	73 (58)	45 (36)	<0.001	7.94 (8.32)	10.4 (8.8)	0.254 (0.7)	Ns
Sometimes	224 (32)	72 (32)	111 (49)	41 (19)	<0.001	54 (24)	136 (60)	34 (15)	<0.001	8.11 (7.42)	6.2 (6.8)	−0.283 (0.64)	Ns
Never	358 (50)	242 (67)	105 (29)	11 (4)	<0.001	209 (58)	134 (37)	15 (5)	<0.001	6.73 (7.26)	2.45 (4)	−0.815 (0.72)	Ns
The distraction improved headache													
Often	159 (23)	83 (52)	52 (33)	24 (15)	Ns	76 (48)	62 (39)	21 (13)	<0.001	5.77 (6.2)	3.06 (4.8)	−0.649 (0.68)	Ns
Sometimes	263 (37)	105 (40)	115 (44)	43 (16)	Ns	96 (37)	125 (48)	42 (16)	<0.001	7.71 (7.9)	5.52 (7.4)	−0.423 (0.79)	Ns
Never	285 (40)	135 (47)	110 (39)	40 (14)	Ns	98 (34)	156 (55)	31 (11)	<0.001	7.21 (7.2)	4.73 (6)	−0.47 (0.82)	Ns

Pt: patients; SD: standard deviation; Ns: not significant (*p* > 0.05); Rho: correlation index of Spearman for association between continuous and numerical covariates; Mwoa: migraine without aura; Mwa: migraine with aura.

### Statistical analysis

The statistical analysis of data was conducted using the open source software R and the suite RStudio. Subjects who did not provide consent to data collection and those whose answers were not analyzable were excluded from statistical analysis.

For the primary objective of analyzing the variation in headache during the lockdown, we considered three primary endpoints. The first primary endpoint was the personal opinion that the subject expressed on the trend of the headache, which could be improved, stable or worsened (trend of headache). The second primary endpoint was the subjective judgment on the variation in the intensity of the attacks, which could be increased, stable or reduced (intensity). The third primary endpoint was the ratio between the frequency of monthly attacks during the lockdown and in the 2 months before the lockdown (frequency).

The first secondary objective was to analyze whether there were differences in trend of headache, and intensity and frequency of attacks between subgroups (patients undergoing prophylaxis and not, chronic and non-chronic patients, and patients from different geographical areas). The second secondary objective was to verify whether the primary endpoints were influenced by psychological aspects (school anxiety, general anxiety, depression, COVID-19 anxiety); the third secondary objective was to analyze possible differences in the psychological factors among patients from areas with different impacts from the COVID-19 emergency (North and South-Central Italy).

The answers to the questionnaire exploring “anxiety” were summarized in synthetic indicators through the “item response theory” technique (11–14). Consequently, each individual was assigned a single score for each dimension (a “COVID-19 anxiety” score, a “general anxiety” score and a “school anxiety” score).

Statistical analysis included two steps. In the first step, a bivariate analysis studied how the different variables (taken singularly) correlated with the primary endpoints. Depending on the nature of the covariates and the response, we considered different statistical tests. For ordinal categorical endpoints (trend of headache and intensity of attacks), we used the Mann-Whitney U-test, the Kruskal-Wallis test and the Spearman’s Rho. For the numeric endpoint (frequency of attacks), we transformed the response on the ratio on the log scale and considered the t-test, analysis of variance (ANOVA) and Spearman’s Rho. In the bivariate analysis, each test was performed individually and did not take into account the contemporary effect of the other covariates.

Multivariate analysis was performed by considering a multiple regression setting on each of the endpoints separately. For ordinal categorial endpoints, we used a generalized linear model (GLM) with cumulative link and proportional odds assumption (15–19). For the frequency ratio endpoint, we considered the Poisson GLM with log link on the frequency reported during the lockdown and the frequency in the 2 months before lockdown as an offset (20–23). First, all the variables that scored a *p*-value ≤0.2 in the bivariate analysis were included in the model. In a second level, we further selected the best subset of covariates through the AIC-based (Akaike information criterion) step forward and backward procedure. Lastly, all the non-significant variables included in the best subset were excluded and the remainder were used to train the final model. The effect of variables in the multivariate analysis was represented by the coefficient $\beta_{i}$. For trend of headache and intensity of attacks, positive values pushed toward improvement, while negative values toward worsening. As for frequency of attacks, negative values indicated a reduction (improvement), while positive values an increase (worsening). For more details of statistical analysis, see Supplemental material).

## Results

We analyzed 707 questionnaires. Demographic and headache features and details of bivariate analysis are reported in Table 1. On average, patients reported that they had been in lockdown for 33 days (from 15–60 days).

### How did headache go?

**Figure 3.**
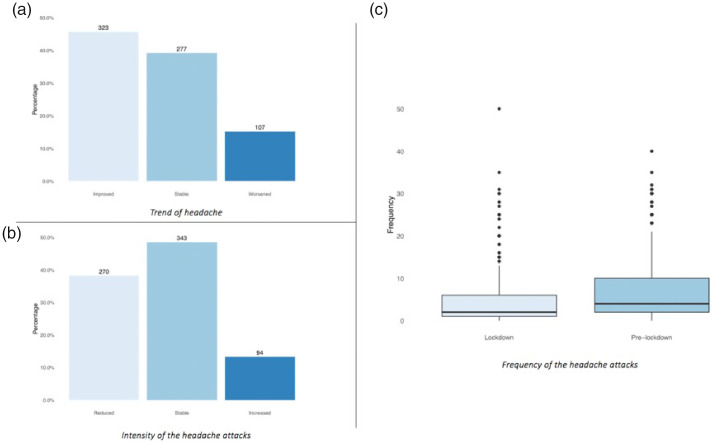
Distribution of patients according to the primary endpoints. (a) trend of headache; (b) intensity of attacks; (c) difference in frequency of attacks between before and during lockdown.

When the patients were asked to express a general opinion on the “trend of the headache*”* during lockdown compared to the previous 2 months, the answers were distributed as follows: 323 patients improved (46%), 277 remained stable (39%) and 107 worsened (15%) (Figure 3(a)). However, stable patients were more likely to have decreased intensity or frequency of attacks rather than an increase (rho 0.73; *p* < 0.0001). Regarding the intensity of the attacks, 270 patients (38%) reported that the intensity of their headache attacks decreased, compared to the intensity before lockdown. In 343 patients (49%) intensity was stable, while it worsened in only 94 (13%) (Figure 3(b)). Concerning the frequency of the attacks, patients reported a mean of 7.38 attacks per month in the 2 months before lockdown. During lockdown, the average monthly number of attacks dropped to 5.4 (Figure 3(c)). The t-test showed that the log ratio was significantly lower than zero (reduction in frequency during lockdown) with *p* < 0.0001 and a 95% confidence interval (CI) between −0.51 and 0.39 (ratio included between 0.60 and −0.67).

We found a significant relationship between age and primary endpoints. In particular, the probability of having a worsening of the trend of headache (rho 0.15; *p* < 0.0001), and intensity (rho 0.18; *p* < 0.0001) and frequency (rho 0.2; *p* < 0.0001) of the attacks increased with increasing age. Male patients presented an increased probability of improving trend of headache (*p* < 0.0001), and intensity (*p* < 0.0001) and frequency (*p* < 0.0006) of attacks during lockdown.

### Features of headache

Although our population was mostly composed of migraine patients, the improvement in headache observed during the lockdown involved both migraine and tension-type headache patients without any significant difference (*p* > 0.05).

The average duration of headache was 39.6 ± 32 months for the total population. We found that patients with a longer history of headache disorders experienced a lower improvement in the trend of headache (rho 0.14; *p* < 0.001), and intensity (rho 0.13; *p* < 0.0006) and frequency (rho 0.17; *p* < 0.0001) of the attacks.

A frequency higher than 15 headaches per month in the 2 months before the lockdown was found in 117/707 patients (16% of the whole population) who had a diagnosis of possible chronic headache disease. Both the trend of headache (*p* > 0.05) and intensity of the attacks (*p* > 0.05) during the lockdown did not depend on whether the patient was chronic or not. In patients with chronic headache disorders, frequency of attacks was reduced on average by nine attacks per month (95% CI between −7 and −11; *p* < 0.01). Frequency calculated by log ratio confirmed that chronic patients with headache disease presented a higher improvement than episodic ones (*p* < 0.001).

### Therapy for headache disorders

As symptomatic drugs, our patients took nonsteroidal anti-inflammatory drugs (80%) and triptans (20%). Patients reported that they took a mean of 4.8 symptomatic drugs (range 0–30) per month prior to lockdown. During lockdown, the monthly drug intake dropped to 2.1 (*p* < 0.01).

Prophylaxis therapy was taken by 104 patients (14%) during the lockdown period. These drugs included amitriptyline (23/104), flunarizine (25/104), topiramate (21/104), valproate (5/104), and nutraceutics (30/104). Forty-four patients (6%) had stopped prophylaxis therapy just before lockdown (within 4 weeks). In order to verify whether headache improvement in patients taking prophylactic therapy could depend on the pharmacological treatment, the primary endpoints were compared between patients “under prophylaxis” and “without prophylaxis” (Table 2). We did not find a significant difference in trend of headache between the groups (*p* > 0.05). This means that the subjective feeling about one’s own headache course during lockdown did not depend on the prophylaxis. The reduction of intensity of attacks was less frequent in patients with than without prophylaxis (24% vs. 41%), while a stable intensity was more often reported by patients with than without prophylaxis (47% vs. 37%) (*p* < 0.05). Regarding the frequency of the attacks, we found that there was no statistically significant difference between patients exposed and not exposed to prophylaxis (−0.84 vs. −1.0; *p* > 0.05). No relationship was found between recently discontinuing prophylaxis and headache worsening during lockdown (trend of headache: *p* > 0.05; intensity of the attacks: *p* > 0.05; frequency of the attacks: *p* > 0.05). We also found that patients who underwent prophylaxis showed higher pre-lockdown drug intake (6.7 vs. 4.5; *p* < 0.0001) and fewer positive coping abilities (*p* < 0.05) than the others.

**Table 2.** Differences between patients under prophylaxis and without prophylaxis in the bivariate analysis.

Prophylactic treatment	Yes 104 (15%)	No 603 (85%)	*p*-value
Mean (SD)			
*Age in years*	13.3 (3.1)	12.4 (3.5)	Ns
Number (%)			
*Sex*			Ns
Female	71 (68)	354 (59)	
Male	33 (32)	249 (41)	
Number (%)			
*Type of headache*			Ns
Episodic migraine without aura	41 (39)	324 (54)	
Episodic migraine with aura	3 (3)	24 (4)	
Tension-type headache	28 (27)	170 (28)	
Chronic migraine	32 (31)	85 (14)	
Number (%)			
*Trend of headache*	38 (37)	285 (47)	Ns
Improved	49 (47)	228 (38)	
Stable	17 (16)	90 (15)	
Worsened			
Number (%)			
*Intensity of attacks*	25 (24)	245 (41)	<0.001
Lower	60 (58)	283 (47)	
Stable	19 (18)	75 (12)	
Higher			
Number (%)			
*Coping ability*	39 (38)	319 (53)	<0.05
High	41 (39)	183 (30)	
Quite	24 (23)	101 (17)	
Low			
Number (%)			
*Deflected mood*			Ns
Never	45 (43)	257 (43)	
Sometimes	47 (45)	309 (51)	
Often	12 (12)	37 (6)	
Mean (SD)			
*Duration of headache since onset, months*	44.4 (37.8)	38.7 (30.7)	Ns
Mean (SD)			
*Number of attacks per month before lockdown*	11.4 (9.25)	6.68 (6.97)	<0.001
Mean (SD)			
*Number of attacks per month during lockdown*	7.48 (7.53)	4.64 (6.53)	<0.001
Mean (SD)			
*Number of drugs per attack intake before lockdown*	6.7 (6)	4.5 (4.8)	<0.001
Mean (SD)			
*Number of drugs per attack intake during lockdown*	3.6 (4.1)	2.6 (4.3)	<0.001
Mean (SD)			
*Severity score of headache pre-lockdown*	9.18 (6.85)	5.6 (5.38)	<0.001
Mean (SD)			
*School anxiety score*	0.236 (0.7)	0.101 (0.7)	Ns
Mean (SD)			
*General anxiety score*	0.04 (0.87)	0.05 (0.8)	Ns

SD: standard deviation; Ns: not significant.

Note: The *p* value refers to the significance of the integrated statistical analysis with which each variable (in italics) was compared between the two groups (yes and no prophylaxis). In particular, the Mann–Whitney U test was used for nominal variables while the Chi-Square test was used for ordinal variables. To facilitate the interpretation of significance we have added comparison of the within-group percentages in the two central columns.

### Effect of psychological factors on the course of headache disorders

Among all respondents, 88% declared that they were taking lessons electronically. About 50% of patients reported a reduction in school effort with the transition from traditional school method to telematic mode. The correlation between reduction of school effort and improvement of the trend of headache was significant (rho 0.15; *p* < 0.001). Furthermore, the reduced effort showed a high correlation with the reduction of frequency (rho 0.035; *p* < 0.001) and intensity (rho 0.15; *p* < 0.001) of the attacks. Regarding anxiety correlated with telematic lessons, we found that the greater the anxiety, the higher was the possibility of headache worsening (trend of headache: rho 0.4, tau 0.35, *p* < 0.001; frequency of attacks: rho 0.36, tau 0.36, *p* < 0.001; intensity of attacks: rho 0.24, tau 0.18, *p* < 0.001). Depressed mood correlated with worsening of trend of headache (rho 0.13; *p* < 0.001), and intensity (rho 0.15, tau 0.14, *p* < 0.001) and frequency (rho 0.04; *p* < 0.001) of the attacks. We found no significant differences in depression or anxiety score in patients from different geographic areas (*p* > 0.05). We found a significant positive correlation between general anxiety and worsening of trend of headache (rho 0.15; *p* < 0.001), and frequency (rho 0.6; *p* < 0.001) and intensity (rho 0.15; *p* < 0.001) of the attacks. We did not find a correlation between COVID-19 anxiety scores and the trend of headache (rho 0.03; *p* > 0.05), and intensity (rho 0.02; tau 0.01; *p* > 0.05) and frequency (rho 0.02; tau 0.01; *p* > 0.05) of the attacks. There were no significant differences in COVID-19 anxiety scores in patients from different geographic areas (*p* > 0.05).

Twenty-three patients reported having had contact with people who tested positive for the nasopharyngeal swab for COVID 19. Of these, only two were family members living together and therefore subjected to real quarantine measures. Forty two percent of these patients came from northern Italy, 33% from central Italy and the remaining 25% from the south. In the group of patients with a history of contact with COVID-19 positive subjects we found no data in favor of greater COVID-19 related anxiety than the remaining population. None of the analyzed subjects was affected by COVID 19 infection.

### Multivariate analysis

**Figure 4.**
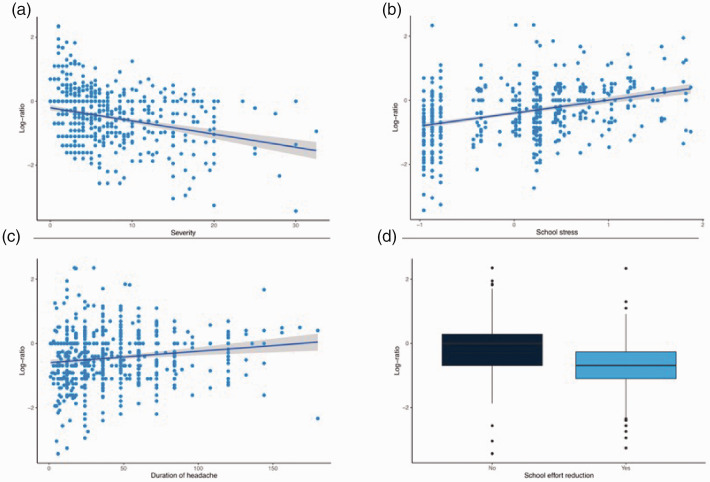
Results from multivariate analysis. Relations between frequency log ratio and severity score (a), duration of headache in months (b), school anxiety (c) and reduction of school effort (d).

Results of multivariate analysis are provided in Figure 4 and Table 3. Reduced school effort improved headache (*p* < 0.0001), while school anxiety (*p* < 0.0001) and the duration of the headache (*p* < 0.0001) favored headache worsening. The severity of the headache before the lockdown was associated with an improvement in the trend of headache and intensity of the attacks (*p* < 0.0001).

**Table 3.** Results of multivariate analysis. Referring to trend of headache and intensity of the attacks, the model intercepts as standard the patients with mean severity and duration of headache, mean school stress and anxiety, no reduction of school effort and without prophylactic drugs. Negative values of coefficients on the log scale (between 0 and 1 on the standard scale) correspond to worsening effect of the variable on the endpoint. Positive values correspond to improvement of the endpoints. Referring to frequency of attacks, we considered the ratio between the monthly rate of attacks observed during the lockdown and the baseline (2 months before the lockdown). Negative values correspond to a reducing effect of the variable on the endpoint, while positive values correspond to an increasing effect of the variable on frequency of the attacks.

Primary endpoint (Significant variable)	Results (β-value ± standard error)	e^β^	Significance
Trend of headache (improved, stable, worsened)			
Effect of covariates			
– Severity	0.077 ± 0.015	1.08	<0.001
– Duration of headache	−0.008 ± 0.002	0.993	<0.001
– Prophylactic treatment	−0.548 ± 0.217	0.578	0.010
– Reduction of school effort	1.275 ± 0.159	3.58	<0.001
– School anxiety	−1.241 ± 0.118	0.289	<0.001
Intensity of the attacks (reduced, stable, increased)			
Effect of covariates			
– Duration of headache	−0.006 ± 0.002	0.994	0.022
– Prophylactic treatment	−0.677 ± 0.212	0.508	0.001
– Reduction of school effort	1.403 ± 0.161	4.066	<0.001
– School anxiety	−0.848 ± 0.113	0.428	0.008
– General anxiety	−0.257 ± 0.097	0.774	<0.001
Frequency of the attacks for month (ratio during/before lockdown)			
Effect of covariates			
– Severity	−0.027 ± 0.002	0.973	<0.001
– Duration of headache	0.001 ± 0.001	1.001	0.012
– Reduction of school effort	−0.557 ± 0.036	0.573	<0.001
– School anxiety	0.342 ± 0.024	1.408	<0.001
– Depressed mood	0.078 ± 0.037	0.925	0.035
– Age	0.033 ± 0.006	1.034	<0.001
– Male sex	−0.104 ± 0.039	0.901	0.008

Patients under prophylactic therapy showed a worsening of both the trend of headache and intensity of attacks (*p* < 0.05). Worsened intensity of attacks was significantly associated with generalized anxiety (*p* < 0.05), and worsened frequency of attacks with depressed mood (*p* < 0.05).

## Discussion

The study showed a significant improvement in the trend of headache, and intensity and frequency of attacks during the lockdown compared to the previous 2 months. Headache improvement was strongly correlated with the reduction of school anxiety and school effort.

The involvement of several headache centers scattered throughout the country made it possible to analyze questionnaire data from regions with different COVID-19 impact. In our patients, trend of headache was not influenced by either COVID-19 anxiety or, in spite of the different geographic impact of the COVID-19 emergency, the geographic origin of our patients.

### Usefulness of prophylactic treatment

A very important finding of the present study is that headache improved in both patients undergoing prophylaxis and those without prophylactic drugs, thus suggesting that the pharmacological treatment did not impact headache course. In the multivariate analysis, we found that prophylactic treatment was even associated with a worsened trend of headache and intensity of attacks. However, this finding does not mean that prophylactic drugs had a negative effect on headache, since it is probably biased by the fact that pharmacological prophylaxis was used in patients with a more severe phenotype. We found that patients adopting prophylaxis had higher pre-lockdown drug intake (6.7 vs. 4.5; *p* < 0.0001) and fewer positive coping abilities (*p* < 0.05), compared with those without pharmacological prophylactic treatment.

The real usefulness of prophylactic drugs has been recently challenged by a randomized controlled study, which failed to demonstrate any superiority of either topiramate or amitriptyline compared to placebo (24). Our study, though not designed to investigate the efficacy of pharmacological prophylaxis, underlines that lifestyle changes related to the lockdown could explain headache improvement in our patients more than any prophylactic drug currently available for the pediatric age.

### Episodic vs. chronic patients

Headache improvement also involved 49% of patients with chronic headache. More generally, the higher the number of attacks and use of symptomatic drugs in the 2 months before the lockdown, the more the headache improved during the lockdown. This is a surprising result, since patients with chronic headache disorders are usually characterized by worse outcome, greater risk of disability, drug resistance and drug abuse (25). Since the determinant that mostly explained headache improvement in our patients was the reduction in school effort and anxiety, the hypothesis can be made that, in turn, anxiety related to school, which is known to increase after the Christmas holidays (4,26), could have contributed to headache severity during the pre-lockdown period (January and February). Studies have shown that the frequency of migraine attacks correlates with the amount of homework and the timing of examinations (27,28). Children with migraine disease have a high rate of school absenteeism, limited extracurricular activities, and difficulties in interpersonal relationships with their peers (29). Other factors, such as loss of leisure time, changes in sleep, and other socio-environmental factors can exert a negative effect on children’s headache during winter (30,31).

### Psychological determinants of headache improvement

In our study, the change in school modalities due to the lockdown played a decisive role in explaining the modification of our patients’ headache disorders. Most of our patients reported a reduction in school effort, although the school had not stopped, but they were required only to attend telematic lessons. It is to be underlined that in the middle of the lockdown the Public Instruction Minister maintained that all students would be admitted to their next class, thus ensuring them a reward independent of their efforts. Interestingly, the patients who showed headache disease worsening during the lockdown were those who kept feeling school stress.

Other psychological factors correlated with headache disorder changes during the lockdown. Generalized anxiety was strongly associated with an increase in both intensity and frequency of the attacks. Although only 7% of the population reported feeling sad, depressed mood correlated with a worsened frequency of attacks. Comorbidity between childhood migraine and psychiatric disorders has been studied extensively (32–34). Depression is one of the most common psychiatric comorbidities in patients with migraine, the relationship between migraine and depression being bidirectional (35). In patients with migraine disease, depression is a significant predictor of migraine evolution into chronic migraine disorder (36). Furthermore, people with migraine disease who have experienced depression are more likely to be refractory to migraine treatments and to develop medication adaptation headache and disability (37,38). As suggested by several studies, anxiety may be a precipitating factor that increases risk for headaches (33,34,39). Additionally, research suggests that some children may be less able to cope with daily life stressors, resulting in an increased number and severity of headaches (40). The association between anxiety and headache disorders could be bidirectional. In children with recurrent headaches, the level of anxiety increases from childhood to adolescence, and a history of headache diseases during childhood increases the risk for anxiety disorders in early adulthood (41).

The results of our study and the improvement in headache seem almost to contrast with the catastrophic and negative atmosphere of the lockdown. Although our work underlines how lifestyle changes have influenced the improvement in headache, other dynamics should also be considered over a longer observation period. In particular, a mechanism that could be involved is resilience, which in several works has been described as decisive for overcoming the difficulties of the lockdown and other catastrophic events and seems to also play a role in the processing of chronic pain (42,43). Resilience is a dynamic and multidimensional construct related to the interactions between individuals and the different environments in which they are experienced (family, peers, school, community and society) (43). Although we did not find correlation between the improvement of headache and emotional impact of the pandemic, we also could not exclude a role of resilient behavior in the management of stress caused by the lockdown.

### Limitations of the study

Our study has some limitations. First, all the analyzed variables were collected through a questionnaire, thus they were less verifiable then data issued from medical records. Although methods for verifying the reliability of the answers have been adopted, we cannot completely exclude that the parents did not answer the questions, even partially, instead of the patients. However, this also reflects clinical practice where, for younger children, even the notations in the diary of headaches and the taking of drugs are recorded by the parents and not directly by the patient. The questionnaire method also implies the limitation of a retrospective analysis of the data collected. Second, in our population, chronic patients and patients undergone prophylaxis represented only a small part of the whole sample. Third, there were considerably fewer patients living in northern Italy than those in the other parts of Italy. Fourth, the results of our study must be interpreted within a first phase of the lockdown when we still had little information available on either the disease or the duration of the measures. Some effects of the lockdown (for example economic and financial ones) were not yet imaginable. Our data must therefore be interpreted in that precise time period. In addition, it was not a purpose of this study to verify a possible direct pathogenic role of the COVID-19 infection on the onset or course of the headache. Neurological symptoms have been reported during COVID-19 disease (44), including potentially fatal ones. Headache has been reported in the course of COVID-19 infection, but at present there is no definite evidence of a role of the virus in the genesis of headache.

## Conclusions

The lockdown state allowed us to study how lifestyle changes could affect the course of headache in a large population of children and adolescents scattered across our country. The most important result was that during the lockdown there was a significant improvement in the subjective trend of headache, and a reduction in the intensity and frequency of the attacks. What mostly explained the headache improvement was the reduction of stress related to school. The improvement was independent of geographic area of origin and pharmacological prophylaxis. All these elements suggest that in children and adolescents, lifestyle is a strong determinant of headache course. This should be taken into account when proposing any treatment for migraine and tension-type headache, letting the patient and her/his parents be aware that any intervention on stress factors is more likely to be effective than the currently available drugs.

## Clinical implications

- During the lockdown, the change in lifestyle and in particular the reduction of school stress led to a significant improvement in headache.
- The improvement in headache has also affected people with chronic headache who may have greater drug resistance.
- The improvement occurred independently of the use of prophylaxis therapy.
- The intervention in lifestyle and stress management represented fundamental elements for the management of headache in children.

## Supplemental Material

sj-pdf-1-cep-10.1177_0333102420965139 - Supplemental material for I stay at home with headache. A survey to investigate how the lockdown for COVID-19 impacted on headache in Italian children Click here for additional data file. Supplemental material, sj-pdf-1-cep-10.1177_0333102420965139 for I stay at home with headache. A survey to investigate how the lockdown for COVID-19 impacted on headache in Italian children by Laura Papetti, Pierfrancesco Alaimo Di Loro, Samuela Tarantino, Licia Grazzi, Vincenzo Guidetti, Pasquale Parisi, Vincenzo Raieli, Vittorio Sciruicchio, Cristiano Termine, Irene Toldo, Elisabetta Tozzi, Paola Verdecchia, Marco Carotenuto, Matteo Battisti, Angela Celi, Daniela D'Agnano, Noemi Faedda, Michela AN Ferilli, Giovanni Grillo, Giulia Natalucci, Agnese Onofri, Maria Federica Pelizza, Fabiana Ursitti, Michelangelo Vasta, Margherita Velardi, Martina Balestri, Romina Moavero, Federico Vigevano and, Massimiliano Valeriani; on behalf of the Italian Headache Society (SISC) specific interest group on pediatric headaches in Cephalalgia
