# Supplementary material for: I stay at home with headache. A survey to investigate how the lockdown for COVID-19 impacted on headache in Italian children
Source: Cephalalgia. 2020 Nov 4;40(13):1459–73. doi: 10.1177/0333102420965139 (PMC7684684; doi:10.1177/0333102420965139)
Supplement: sj-pdf-1-cep-10.1177_0333102420965139 - Supplemental material for I stay at home with headache. A survey to investigate how the lockdown for COVID-19 impacted on headache in Italian children [file sj-pdf-1-cep-10.1177_0333102420965139.pdf]

Questionnaire structure

The questionnaire was built by neuropediatricians and psychologists of Bambino Gesù Children Hospital, expert in pediatric headaches. It was reviewed and approved by eight experts on pediatric headache belonging to the Italian Society for the Study of Headache (SISC).
Questionnaire feasibility was tested on a group of 10 pediatric patients and their families, in order to verify the clarity of questions and answers and the compilation time (about 30 minutes). Then, it was sent to 900 families.
Questionnaire design allowed patients older than 13 years to fill it alone and youngest children to fill it with the support of one parent. Except in a few specific cases, closed-ended questions were included.
The questionnaire was preceded by a description of the aims of the study and an information notice for parents and the child. The access to the questions was possible only after consenting to privacy, storage and data management.
Forty-seven questions addressed directly to patients and sixteen to their parents. Questions were sorted to allow a gradual transition across 12 topics.
Topics are summarized as follows:

Questions for the patients (possible help from the parents):

1. *Demographic features:* sex, age, town of residence.
2. *Lockdown information:* first day of lockdown (day and month), compliance with lockdown regulations, exposure to COVID-19 cases or cases of COVID-19 disease in the family and quarantine measures. Patients were asked about the persons with whom they spent their free time (parents, grandparents, babysitters, siblings).
3. *Features of headache:* patients were asked: I) the duration of the headache in months from the first attack, II) a general judgment on the *"trend of the headache"* during the lock down (possible answers included: "improved", "stable" or "worsened", III) change in the intensity of the attacks (possible answers: "lower", "stable", "higher"), and IV) the number of attacks per month in the two months before the lockdown and during the lockdown.
4. *Therapy for headache.* One question asked the number of monthly assumptions of drugs for the attack (nonsteroidal anti-inflammatory drugs or triptans) in the two months preceding the lockdown and during the lockdown. Moreover, patients were asked if they were assuming or had recently assumed drugs for prophylaxis (flunarizine, topiramate, amitriptyline, nutraceutics, valproate, other). Parents were asked to specify the starting and ending date of the therapy. Lastly, patients were asked whether they were having a psychotherapy during the lockdown.
5. *Lifestyle changes:*
   The following aspects were considered:
   a) sleep: mean hours of sleep before and during the lockdown and sleep rhythm (time of falling asleep or awakening, night awakenings). Exposure to electronic devices (average of minutes per day of exposure to tablets, smartphones, computers) before and during lockdown.
   b) dietary style : patients were asked whether they had significantly changed the type of diet and the changes consisted of: increase/decrease in proteins and/or carbohydrates and/or fats and/or fruit or vegetables and/or sweets/carbonated drinks.
6. *Physical activity:* we asked if the patients continued or suspended their physical activity, if they were doing physical activity at home and what type of activity
7. *Stress and anxiety about COVID-19:* a group of questions explored the presence of elements of anxiety in relation to the information circulating on COVID-19. We asked patients: 1) if after hearing news on COVID-19, they experienced symptoms such as sore throat, head or fever; 2) if their headaches worsened when they heard about the virus, 3) if they asked parents for information about COVID-19; 4) if they felt the need for reassurance about their symptoms; 5) if during the lockdown they happened to think that they would never have a life like before. Possible answers to these questions included: always, often, sometimes, never.
8. *General Mood.* Two questions explored the general mood in the weeks of lockdown. We asked the patients if they happened to feel sad or nervous or tense and if they felt unable to control their concerns and fear that something terrible might have happened (often, sometimes, never). The questions that explored anxiety, stress and mood were elaborated on the basis of GAD-7 (Generalized Anxiety Disorder 7-item scale) and PHQ9 (Patient Health Questionnaire 9-item scale) for comorbidity with anxiety and mood disorder, respectively..
9. *School Anxiety.* In this section we firstly asked the patients if they were following the school lessons electronically or not. Then, patients were asked whether their headache worsened when they followed the online lessons or did homeworks and whether they asked the parents about the re-start of the traditional school attendance (always, often, sometimes, never). Lastly, we asked the patients if they had the feeling of a reduction in school effort (yes, no, I don't have the impression that the activities are reduced).

Questions for the parents:

1. *Lockdown informations*: compliance with lockdown regulations, exposure to COVID-19 cases or cases of COVID-19 disease in the family and quarantine measures.
2. *Features of headache:* type of headache (according to the diagnosis received by the reference headache center).

3. *Information regarding the living place:* we asked: i) the number of family members with whom the lockdown was shared and the number of rooms in the home; ii) to indicate whether the house had outdoor spaces such as a livable garden or terrace; iii) whether or not the parents were in smart working at home.
4. *Stress and anxiety about COVID-19*: we asked parents if their child needed COVID-19 health insurance and if he asked for information on COVID-19 news.
5. *School Anxiety:* We asked parents if telematics lessons caused headaches in their child and if they feared that a long stay in front of the computer for the lessons could make their child's headache worsen. Possible answers included always, often, sometimes, never.
6. *Coping.* Two questions addressed to parents explored the child's coping ability in relation to headache: we asked if during the lockdown their child seemed to be less disturbed by headache in his activities and if their child's headache improved if he kept engaged in some activity (i.e. online school, physical activity). Possible answers included always, often, sometimes, never.
7. *Somatization.* Lastly, the parents were asked if their child was developing new symptoms without a clear objective sign and what kind of symptoms (for example pain in other locations, coughing, malaise, dizziness).

Details of statistical analysis.

Missing values management. Some subjects left some items unanswered. If the unanswered item was one of the primary endpoints ("intensity of the headache attacks during lockdown", "frequency of the attacks before lockdown" and "frequency of the attacks during lockdown") the record was eliminated. All the missing values referred to any other item have been imputed using the general average (numeric variable) or the most frequent value (categorial variables).

Multivariate Models. For the trend of headache and intensity of the attacks (ordinal endpoints) we considered the generalized logistic model with cumulative link and proportional odds assumption. Let Y be the endpoint characterized by the three ordered categories 1, 2 and 3 (improved, stable, worsened or reduced, stable, increased) and $x_1, x_2, \ldots, x_p$ the set of p covariates, we modelled the log-odds ratios of observing a response lower or equal to 1 and the log-odds ratio of observing a response lower or equal than 2 in the following linear fashion:

$$\log \frac{P(Y \leq 1|X)}{P(Y > 1|X)} = \beta_{01} + \beta_1 * x_1 + \cdots + \beta_p * x_p$$

$$\log \frac{P(Y \leq 2|X)}{P(Y > 2|X)} = \beta_{02} + \beta_1 * x_1 + \cdots + \beta_p * x_p$$

The intercepts β_{01} and β_{02} represented the value of the log-odds for the reference individual (all covariates equal to 0). The effect of each covariate x_i was linear with coefficient β_i on the log-odds, which corresponded to a multiplicative effect of $e^{\beta_i x_i}$ on the linear scale.
Negative values of the coefficient β_i corresponded to a decreasing effect of x_i on the estimated probabilities of observing low values on the scale (the covariate was pushing toward a "worse" condition); positive values of the coefficient β_i corresponded to an increasing effect of x_i on the estimated probabilities of observing low values on the scale (the covariate was pushing toward an “improved" condition).
This model was trained in the software R using the polr function of the MASS package.

For the ratio of frequency of the attacks, since the single frequency was a count and not a continuous variable, we considered the Poisson GLM with log link on the frequency reported during the lockdown and the frequency in the two months before lockdown as an offset. Let Y_d be the frequency reported during lockdown, Y_b be the frequency reported for the two months before the lockdown, and $x_1, x_2, \ldots, x_p$ the set of p covariates, we modelled Y_d as a Poisson with rate parameter θ_d and included Y_b as an offset, so that:

$$\log \theta_d = \log Y_b + \beta_0 + \beta_1 * x_1 + \cdots + \beta_p * x_p$$

$$\log \frac{\theta_d}{Y_b} = \beta_0 + \beta_1 * x_1 + \cdots + \beta_p * x_p$$

The intercept β_0 represented the variation of the log rate (monthly) for the reference individual (all covariates equal to 0). The effect of each covariate x_i was linear with coefficient β_i on the log-rate, which corresponded to a multiplicative effect of $e^{\beta_i x_i}$ on the linear scale. Negative values of the coefficient β_i corresponded to a decreasing effect of x_i on the ratio between the estimated rate during lockdown and the frequency reported before the lockdown (the covariate was pushing toward an “improved" condition); positive values of the coefficient β_i corresponded to an

increasing effect of x_i on the ratio between the estimated rate during lockdown and the frequency reported before the lockdown (the covariate was pushing toward a “worsened" condition).
The model was trained in R using the GLM function available in the stats package.
Best subset selection of the covariates was performed through the AIC-based step forward and backward procedure using the stepAIC function from the MASS package.

Before fitting any of the following models, all the numerical variables were centered on their average value, so that a value of 0 of the covariate would have corresponded to the average individual. For the categorical variables, one category was always chosen as baseline and the model estimated the effect as a variation from such a baseline.

Interpretation of multivariate analysis.
Trend of headache.
The model intercepts represent the condition of the reference individual, considered as a subject with average severity, average duration of headache, average school anxiety, no prophylactic treatment and no reduction of school effort.
The estimated values of $\beta_{01} = -0.848$ and $\beta_{02} = 1.651$, reported on the linear scale ($e^{\beta} = 0.428, 5.207$) indicate the probabilities to assume low values of the scale compared to high values of the scale (probability to improve is 0.4 times that to be stable or worsened; probability to improve or be stable is 5 times that to be worsened).
Converting it in terms of probabilities of the single categories, this leads to a probability of having an improving trend of 0.3, stable 0.54 and worse 0.16 for the reference individual.
The effect of the variables included in the best subset model on the log odds ratios is represented by the coefficients β_i, which pushes toward improvement for positive values and worsening for negative values: severity of headache pre-lockdown (β = 0.077 ± 0.016; p<0.0001); duration of headache (β = -0.008 ± 0.002; p<0.0001); prophylactic treatment (β = -0.548 ± 0.217; p= 0.011); reduction of school effort (β = 1.275 ± 0.217; p< 0.0001); school anxiety (β = -1.241 ± 0.118; p< 0.0001). On the linear scale (**e**$^{\beta}$), the values represent the multiplicative factor by which the probability of assuming low values of the scale are multiplied in case of a unitary variation of the respective covariate (for numeric variables) or by assuming the specified modality (for categorical variables). For instance, a unitary increment of the pre-lockdown severity leads to probabilities of assuming low values multiplied by 1.08 (increased), while the presence of prophylactic treatment (absent in the baseline) leads to probabilities of assuming low values multiplied by 0.578 (decreased).
Intensity of headache.
The model intercepts represent the condition of the reference individual, represented by a subject with average duration of headache, average school anxiety , no prophylactic treatment, no reduction of school effort and average general anxiety.
The estimated values of $\beta_{01} = -1.245$ and $\beta_{02} = 1.711$, reported on the linear scale ($e^{\beta} = 0.287, 5.534$) indicate the probabilities to assume low values of the scale compared to high values (probability of reduced intensity is 0.28 times that to be stable or worsened; probability of reduced or stable intensity is 5.5 times that of increased intensity).
Converting it in terms of probabilities of the single categories, this leads to a probability of reduced intensity of 0.224, stable intensity of 0.623 and increased intensity of 0.153 for the reference individual.
The effect of the variables included in the best subset model on the log odds ratios is represented by the coefficients β_i, which pushes toward reduction of intensity for positive values and increasing of intensity for negative values: duration of headache (β = -0.006 ± 0.002; p=0.022); prophylactic treatment (β = -0.677 ± 0.212; p=0.001); reduction of school effort (β = 1.403 ± 0.161; p<0.0001); school anxiety (β = -0.848 ± 0.113; p=0.008) ; general anxiety (β = -0.257 ± 0.097; p<0.0001).
On the linear scale (**e**$^{\beta}$), the values represent the multiplicative factor by which the probability of assuming low values of the scale are multiplied in case of a unitary variation of the respective covariate (for numeric variables) or by assuming the specified modality (for categorical variables). For instance, a unitary increment in duration of headache leads to probabilities of assuming low values multiplied by 0.994 (decreased), while the reduction of school effort (absent in the baseline) leads to probabilities of assuming low values multiplied by 1.403 (increased).
Frequency of headache.
The model intercept represent the condition of the reference individual, represented by a female subject with average age, average severity, average duration of headache, average school anxiety, no reduction of school effort and with no depressed mood.
The estimated value of β_0=-0.169, reported on the linear scale ($e^{\beta} = 0.82$) indicate the proportion of the pre-lockdown headache rate experienced by the reference individual during the lockdown (rate during lockdown is the 82% of the rate before lockdown).
The effect of the variables included in the best subset model on the log rate is represented by the coefficients β_i, which pushes toward a reduced rate for negative values and an increased rate for positive values: sex is male (β = -0.104 ± 0.039; p=0.008); age (β = 0.033 ± 0.006; p<0.0001); severity (β = -0.027 ± 0.002; p<0.0001); duration of headache (β = 0.001 ± 0.001; p=0.012); school anxiety (β = 0.342 ± 0.024; p<0.0001); reduction of school effort (β = -0.557 ± 0.036; p<0.0001);
depressed mood (β=0.078±0.0037;p=0.035).

On the linear scale ($\mathbf{e}^{\beta}$), the values represent the multiplicative factor by which the ratio between the lockdown frequency and the pre-lockdown frequency is multiplied in case of a unitary variation of the respective covariate (for numeric variables) or by assuming the specified modality (for categorical variables). For instance, a unitary increment of the school anxiety leads to a ratio multiplied by 1.408 (increased), while the presence of a reduction of school effort (absent in the baseline) leads to a ratio multiplied by 0.573 (reduced).
